# Diabetic Ketoacidosis With Acute Pancreatitis in Patients With Type 2 Diabetes in the Emergency Department: A Retrospective Study

**DOI:** 10.3389/fmed.2022.813083

**Published:** 2022-03-17

**Authors:** Li Ping Ma, Xue Liu, Bei Chen Cui, Yan Liu, Cong Wang, Bin Zhao

**Affiliations:** Department of Emergency, Beijing Jishuitan Hospital, Beijing, China

**Keywords:** acute pancreatitis, diabetic ketoacidosis, risk factors, diabetes mellitus, hyperlipidemia

## Abstract

**Objective:**

This study aims to explore the incidence and clinical features of acute pancreatitis (AP) in patients with type 2 diabetes diabetic ketoacidosis (DKA) in the emergency department and discuss the predictive value of some pathological indicators for AP in DKA.

**Methods:**

Inpatient medical data of DKA patients hospitalized to our hospital's emergency department between January 2017 and January 2021 were evaluated retrospectively. These DKA patients were split into two groups based on whether they had AP or not. We examined the two groups' epidemiologic features, baseline laboratory results, and clinical outcomes. The Bedside Index for Sequential Organ Failure Assessment (SOFA), Acute Physiology and Chronic Health Evaluation II (APACHE II), and Logistic Organ Failure System (LODS) scores were computed and compared across groups.

**Results:**

The prevalence of AP in DKA patients was 15.53%. The difference in Abdominal pain between the two groups of patients was statistically significant (*p* < 0.001), and there was no statistical difference in age, gender, and BMI. The DKA and AP group LOS (*P* < 0.001), ICU admission rate (*P* = 0.046), anion gap (*P* < 0.001), red blood cell (*P* = 0.002), hemoglobin (*P* < 0.001), hematocrit (*P* = 0.002), serum triglyceride (*P* < 0.001), serum cholesterol (*P* < 0.001), serum amylase (*P* = 0.004), random glucose (*P* = 0.028), plasma fibrinogen (*P* < 0.001), glycosylated hemoglobin [HbA1c (%); *P* = 0.008] higher than the DKA group, pH (*P* < 0.001), carbon dioxide combining power (CO2CP; *P* < 0.001), ionized calcium (Ca2+; *P* = 0.022), ionized sodium (Na+; *P* = 0.001), and correction Na (*P* = 0.034) lower than the DKA group. Multivariate analysis showed that low pH (*P* < 0.05), hypertriglyceridemia (*P* = 0.001), and hypercholesterolemia (*P* = 0.01) were risk factors for DKA combined with AP. ROC curve analysis showed that the three cut-off value: serum triglycerides of 10.52 mmol/L, serum cholesterol of 9.03 mmol/L, and pH of 7.214. Serum triglyceride has the largest area under the curve (0.93). Under this cut-off value, the sensitivity (80%) and specificity of serum triglyceride, the degree (93.7%) is the highest, while the positive predictive value (62.0%) and negative predictive value (94.7%) of serum cholesterol are the highest.

**Conclusions:**

A severe episode of DKA with significant acidosis and hyperlipidemia is more likely to be linked with AP. The frequently used critical illness score is ineffective in determining the severity of the condition. When the serum triglyceride cut-off value is 10.52mmol/L, it has a higher predicted value for AP in DKA.

## Introduction

Acute pancreatitis (AP) as a cause or consequence of diabetic ketoacidosis (DKA) has previously been documented (1–3). The diagnosis of AP was based primarily on clinical symptoms and concomitant increases in serum pancreatic enzymes in some of the studies, with no confirming radiographic findings (1–3). Numerous case studies highlighting diabetic coma as an uncommon consequence of severe AP also observed that several of these individuals did not have underlying diabetes (2, 4, 5). These single case reports contributed nothing to our understanding of the true incidence or course of AP in DKA.

As it is well-known, AP and DKA are life-threatening diseases. The co-occurrence ratio of DKA and AP is low in all emergency patients, and both have common clinical features such as abdominal pain and elevated amylase, which are easily ignored by emergency physicians. According to certain research, while more than 40% of DKA patients report abdominal pain on admission, and 17% had significant abdominal pathology mainly AP (6), it is readily missed because of their clinical similarities. This creates significant difficulties for emergency physicians. As a result, this study focused on identifying risk factors for the coexistence of AP in DKA patients in order to aid in early detection.

## Materials and Methods

### Research Subject

We analyzed clinical data from patients presenting to our hospital's emergency department between January 2017 and January 2021. A total of 170 patients with type 2 diabetes mellitus (T2DM) with DKA were admitted to our hospital. According to the occurrence of AP and the informed consent of all patients, the patients were divided into AP group, DKA, and AP group after rechecking the clinical information and diagnosis.

Inclusion criteria were as follows: (1) All patients met the diagnostic criteria for T2DM (7): fasting blood glucose (FBG) levels ≥7.8 mmol/L or 2 h post-prandial blood glucose (2hPBG) levels ≥11.1 mmol/L. Diagnosis of type 2 diabetes according to differential diagnostic criteria for types 1 and 2 diabetes. (2) DKA diagnostic criteria: urine ketone tested to be positive, blood ketone >0.3 mmol/L, and pH value by blood gas analysis <7.35, suggesting metabolic acidosis. (3) AP diagnostic criteria (8): (i) abdominal pain characteristic of AP; (ii) serum lipase or amylase levels that were at least three times the upper limit of the normal range; (iii) characteristic findings of acute pancreatitis on cross-sectional imaging (computed tomography or magnetic resonance imaging) or transabdominal ultrasonography, AP can be diagnosed if two of the above three items are met. (4) Patients signed the informed consent. The relevant biochemical indicators were all the venous blood test results collected within 48 h after admission.

Exclusion criteria were as follows: (1) T1DM. (2) Patients who were <18 years old. (3) Patients who were pregnant. (4) In the absence of comprehensive data profiles or informed consent. Finally, 161 DKA patients were included in this research.

### Data Collection

We collected demographic data (age and gender), body mass index (BMI), symptoms at the time of the visit, and a history of comorbidities (diabetes mellitus, hypertension, hyperlipemia, pulmonary illness, and alcohol intake), as well as treatment consequences. We gathered all laboratory test results. At the time of admission, data were obtained to compute the acute physiology and chronic health assessment II (APACHE II), the Sequential Organ Failure Assessment (SOFA) score, and the Logistic Organ Dysfunction System (LODS).

### Statistical Analysis

For statistical analysis, IBM SPSS Statistics for Windows version 22 was used (IBM Corp., Armonk, NY, USA). For skewed distributions, continuous variables are presented as mean $\bar{x}$ ± *standard deviation (SD*) or median (interquartile range) and were compared using the Student's *t*-test or the Mann–Whitney *U* test, as appropriate. The Pearson χ^2^ test, continuity-adjusted chi-square, and Fisher's exact test were used to compare categorical variables. In previous comparisons between the two groups, multivariate logistical regressional analysis was carried out to examine the factors that contributed to DKA and AP's coexistence in variables with *P* < 0.05. The odds ratios (ORs) and 95% confidence intervals (CIs) for the variables were calculated. Separate subject receiver operating characteristic curves (ROC) were plotted to establish cut-off values and compare sensitivity, specificity, positive predictive value, and negative predictive value. If the *P*-value was <0.05, the result was judged significant.

## Results

The total number of DKA occurrences in diabetes mellitus investigated was 170. One patient was 16 years old, three had type 1 diabetes, and five had incomplete data profiles; these patients were removed. As a result, this study comprised 161 T2DM DKA patients. These DKA patients were divided into two groups based on whether they had AP or not: 136 with DKA only (the DKA group) and 25 with DKA plus AP (the DKA and AP group), with an overall prevalence of 15.53%.

### Clinical Features

Table 1 compares the demographic and clinical characteristics of the two groups at admission. The DKA group had a mean age of 37 (29 and 53) years, with 77 men and 59 women making up the total of 136. The average age in the DKA and AP groups was 30 (26.5, 14.25), with 19 men and 6 women, for a total of 25, with no significant difference (*p* = 0.069) between the two groups. The DKA and AP groups showed similar mean BMI with the DKA group (26.85 vs. 23.41, *p* = 0.126). In terms of alcohol consumption, hypertension, hyperlipidemia history, CHD, diabetes mellitus history, or pulmonary illness, there was no significant difference between the DKA and AP groups. When comparing abdominal discomfort (39.7 vs. 80%, *p* < 0.001), there was a significant difference between the two groups, the DKA group often presented with episodic abdominal pain that was not fixed in place, DKA combined AP group presented with acute, sudden, persistent and severe upper abdominal pain, and some patients radiated to the back. There was no significant difference between the two groups for nausea and vomiting, mental status changes, weakness, fever, dyspnea, and palpitation.

**Table 1:** Comparison of demographic and clinical characteristics of diabetic ketoacidosis (DKA) patients with and without acute pancreatitis (AP) at admission.

	**Total (161)**	**The DKA group (136)**	**The DKA and AP group (25)**	***P***
**Manifestations upon admission**				
Abdominal pain	74 (46.0%)	54 (39.7%)	20 (80%)	<0.001
Nausea and vomitting	106 (65.8%)	89 (65.4%)	17 (68%)	0.804
Mental status changes	22 (13.7%)	20 (14.7%)	2 (8.0%)	0.370
Weakness	34 (21.1%)	32 (23.5%)	2 (8.0%)	0.080
Fever	22 (13.7%)	17 (12.5%)	5 (20%)	0.316
Dyspnea	29 (18%)	27 (19.9%)	2 (8%)	0.257
Palpitation	24 (14.9%)	23 (16.9%)	1 (4%)	0.174
**History of comorbidities**				
Alcohol Consumption	4 (2.5%)	2 (1.5%)	2 (8.0%)	0.219
Hypertension	20 (12.4%)	17 (12.5%)	3 (12%)	>0.999
Hyperlipidemia	15 (9.3%)	14 (10.3%)	1 (4%)	0.535
CHD	8 (5%)	8 (5.9%)	0 (0)	0.360
Diabetes mellitus	94 (58.4%)	81 (59.6%)	13 (52%)	0.480
pulmonary disease	2 (1.3%)	1 (0.7%)	1 (4%)	0.713
**Patients characteristics**				
Gender				0.069
Male	96 (59.6%)	77 (80.2%)	19 (19.8%)	
Female	65 (40.4%)	59 (90.8%)	6 (9.2%)	
Age		37 (29.00, 53.00)	30 (26.50, 14.25)	0.007
BMI		23.41 (21.66, 26.19)	26.85 (22.49, 30.53)	0.126

### Biomedical Profile

A comparison of patients with and without AP in terms of their biochemical profiles. In Table 2, the DKA and AP groups had a higher rate of intensive care unit (ICU) admission (7.4 vs. 20%, *p* < 0.05) and a longer length of stay (LOS) [12 (9 and 14.25) day vs. 14 (13.5 and 20) day, *p* < 0.01]. Each of the two groups has lost one member. There was no statistically significant difference in death rates. Additionally, no statistically significant difference in respiratory distress requiring intubation, Glasgow Coma Scale (GSC), or age-adjusted Charlson Comorbidity Index was observed (aCCI). The bedside index for SOFA score, APACHE II, and LODS were computed and compared between the two groups, but no statistically significant difference was seen. Patients with concurrent AP had lower serum levels of pH (*p* < 0.001), carbon dioxide combining power (CO2CP, *p* < 0.01), ionized calcium (Ca2+, *p* < 0.01), ionized sodium (Na+, *p* < 0.01), correction sodium by blood glucose (*p* < 0.05), and higher levels of anion gap (AG, *p* < 0.01), red blood cell (RBC, *p* < 0.01), hemoglobin (HGB, *p* < 0.01), hematocrit (HCT, *p* < 0.01), serum triglyceride (*p* < 0.01), serum cholesterol (*p* < 0.01), serum amylase (*p* < 0.01), random glucose (*p* < 0.05), plasma fibrinogen (*p* < 0.01), and glycosylated hemoglobin (HbA1c, *p* < 0.01).

**Table 2:** Comparison of clinical and biochemical results in DKA patients with and without AP.

	**The DKA group**	**The DKA and AP group**	***p***
**Hospitalization course**			
LOS (days)	12.00 (9.00, 14.25)	14.00 (13.50, 20)	<0.001
ICU admission	10 (7.4%)	5 (20%)	0.046
Respiratory distress	3 (2.2%)	1 (4%)	>0.999
requiring intubation			
In-hospital mortality	1 (0.7%)	1 (4.0%)	0.710
**Severity scores**
aCCI	1.00 (1.00, 1.00)	1.00 (1.00, 1.00)	0.098
GSC	15.00 (15.00, 15.00)	15.00 (15.00, 15.00)	0.868
APACHE II	6.00 (4.00, 9.00)	6.00 (3.50, 14.00)	0.771
SOFA	0.00 (0.00, 0.00)	0.00 (0.00, 1.00)	0.690
LODS	1.00 (0.00, 3.00)	1.00 (0.00, 2.00)	0.057
**Laboratory**
PH	7.28 (7.22, 7.32)	7.09 (7.05, 7.24)	<0.001
AG (mmol/L)	19.63 ± 5.72	25.3 ± 4.89	<0.001
WBC (× 10^9^/L)	13.86 (9.45, 20.03)	15.03 (13.75, 20.10)	0.122
RBC (×10^9^/L)	5.05 (4.63, 5.66)	5.57 (5.33, 5.82)	0.002
HGB (g/L)	153.29 ± 23.16	173.32 ± 19.30	<0.001
HCT (%)	43.84 ± 5.87	47.66 ± 4.34	0.002
ALT (U/L)	21.00 (14.00, 30.50)	20.00 (14.50, 32.00)	0.758
AST (U/L)	16.00 (13.00, 25.00)	16.00 (12.50, 23.50)	0.705
TBIL (mmol/L)	13.35 (9.88, 17.83)	13.50 (8.25, 21.20)	0.899
BNU (mmol/L)	6.85 (4.63, 10.90)	7.40 (5.25, 11.20)	0.401
Creatine (umol/L)	69.50 (53.00, 99.00)	80.00 (59.50, 142.00)	0.323
CO_2_CP (mmol/L)	22.00 (14.75, 33.25)	9.00 (7.00, 19.00)	<0.001
Serum triglyceride	2.83 (1.66, 5.11)	23.13 (10.92, 28.80)	<0.001
(mmol/L)			
Serum cholesterol	5.87 (4.76, 7.74)	11.62 (7.85, 15.19)	<0.001
(mmol/L)			
LDH (U/L)	193.50 (170.50, 239.00)	200.00 (161.00, 254.00)	0.603
Serum amylase (U/L)	63.00 (35.75, 242.25)	159.00 (91.00, 760.00)	0.004
Random glucose	27.76 ± 12.20	33.78 ± 13.95	0.028
(mmol/L)			
PT (s)	12.70 (11.83, 13.98)	13.10 (12.15, 14.15)	0.341
APTT (s)	27.65 (23.58, 33.08)	29.30 (27.20, 31.55)	0.408
Plasma fibrinogen	335.15 (254.73, 406.78)	539.60 (332.50, 658.40)	<0.001
(mg/dl)			
D-dimer (mg/L)	0.56 (0.24, 1.15)	0.99 (0.32, 3.07)	0.098
HbA1c (%)	11.60 ± 2.64	13.11 ± 2.09	0.008
Ca^2^^+^ (mmol/L)	2.33 (2.20, 2.46)	2.09 (1.95, 2.41)	0.022
Na^+^ (mmol/L)	134.00 (130.00, 137.00)	130.00 (124.50, 133.50)	0.001
Correction Na^+^ (mmol/L)	140.03 (135.75, 142.80)	136.97 (131.00, 144.46)	0.034

### Multivariate Analysis of Risk Factors

Multiple criteria were used to examine the findings of the laboratory examination following admission, as indicated in Table 3. Low pH, hypertriglyceridemia, and hypercholesterolemia were found to increase the risk of DKA and AP. Because of the restricted pH range, the pH is represented as layers. We discovered that pH <7.25 was statistically significant.

**Table 3:** Multivariate analysis adjusted odds ratios of factors linked with DKA and AP.

	**β**	**S.E**.	**Wald**	***p***	**Adjusted OR (95% CI)**
PH (7.251–7.350)			13.152	0.004	
PH (7.051–7.150)	5.345	1.669	10.255	0.001	209.551 (7.953–5521.297)
PH (7.151–7.250)	2.964	1.162	6.508	0.011	19.380 (1.987–188.983)
Serum triglyceride (mmol/L)	0.205	0.059	11.929	0.001	1.227 (1.092–1.378)
Serum cholesterol (mmol/L)	0.351	0.137	6.587	0.010	1.421 (1.087–1.858)

### The Predictive Value of Various Indicators

**Figure 1:**
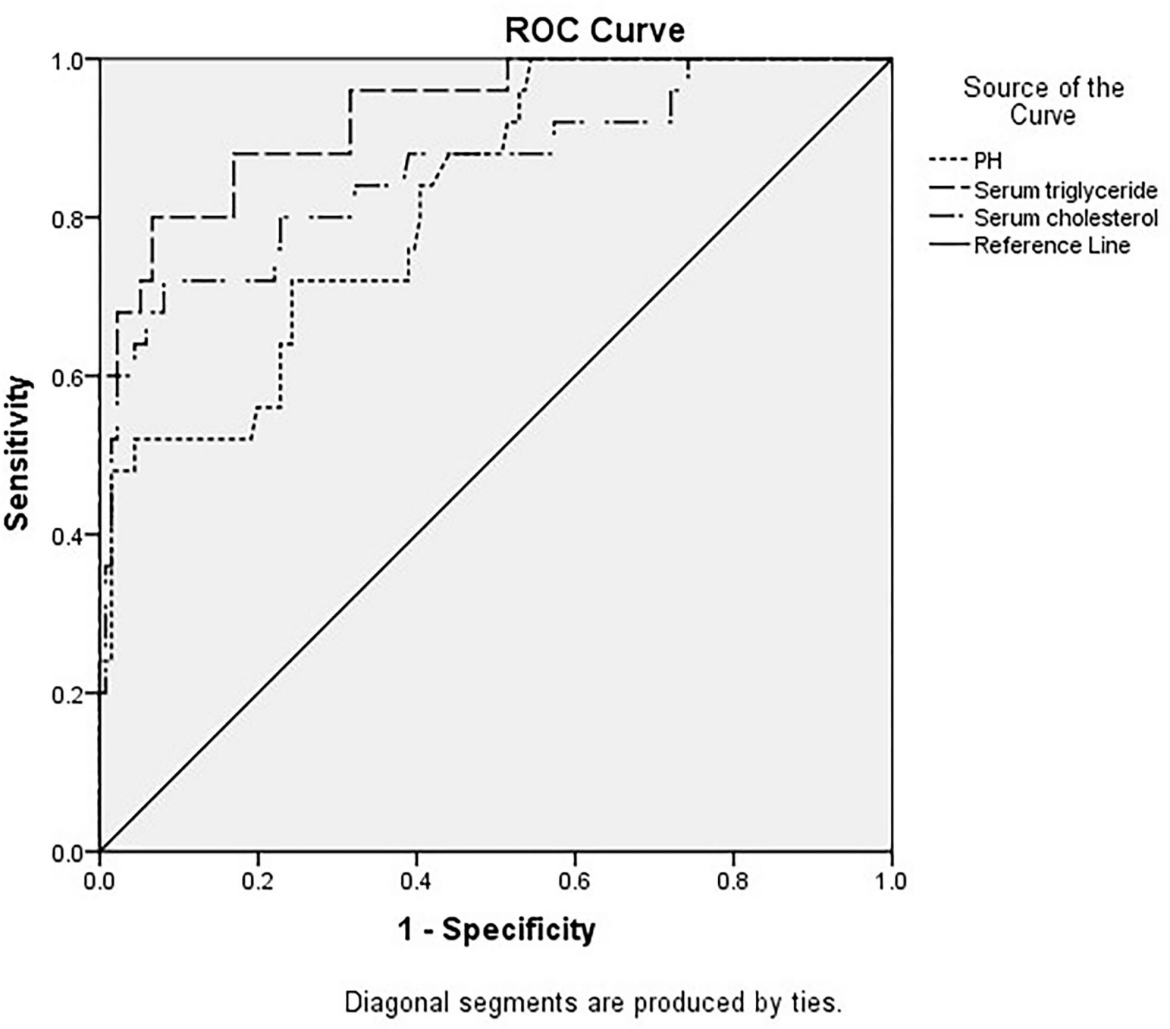
Different indicators predict the receiver operating characteristic (ROC) curve of diabetic ketoacidosis (DKA) patients with acute pancreatitis (AP).

The areas under the curve of serum triglycerides (right of Figure 1), serum cholesterol (right of Figure 1), and pH (left of Figure 1) for predicting AP in DKA patients with type 2 diabetes were 0.93 (95% CI 0.875–0.985), 0.86 (95% CI 0.768–0.951), and 0.821 (95% CI 0.737–0.905) in Figure 1 and Table 4. In Table 5, ROC curve analysis is performed to estimate the sensitivity, specificity, positive predictive value, and negative predictive value of type 2 diabetic DKA patients with AP with serum triglycerides of 10.52 mmol/L, serum cholesterol of 9.03 mmol/L, and pH of 7.214.

**Table 4:** Different indicators for the prediction of the DKA patients with AP.

**Test result variable(s)**	**Area**	**Std. Errors**	***P***	**Asymptotic 95% confidence interval**	
				**Lower bound**	**Upper bound**
Serum triglyceride	0.930	0.028	<0.001	0.875	0.985
Serum cholesterol	0.860	0.047	<0.001	0.768	0.951
PH	0.821	0.043	<0.001	0.737	0.905

**Table 5:** The predictive value of different indicators for DKA combined AP.

**Variable**	**CUT-OFF**	**Sensitivity**	**Specificity**	**Positive predictive value (PPV)%**	**Negative predictive value (NPV)%**
Serum triglyceride (mmol/L)	10.52	80%	93.2%	60.7%	94%
Serum cholesterol (mmol/L)	9.03	72%	91.7%	62.0%	94.7%
PH	7.214	75.7%	72.0%	42.7%	93.5%

## Discussion

Diabetic ketoacidosis (DKA), also known as diabetic acidosis or diabetic coma, is a serious complication of type 2 diabetes. In our single-center retrospective analysis, we found AP in 25 (15.53%) of 161 DKA patients, and another study showed that the incidence of AP in DKA patients was about 11% (9), which is slightly lower. In this study, there may be differences in the diagnostic procedures and standards between the two studies, as well as the update of laboratories and imaging equipment.

Over the past six decades, the problem of DKA merging with AP has been identified and has received widespread attention. At present, imaging is consistent with changes in pancreatitis, and serum amylase levels exceed the upper limit of the normal value by 3 times. Persistent abdominal pain in the upper abdomen is often used to diagnose acute pancreatitis, but abdominal pain and hyperamylasemia may exist in many patients with DKA, making it potentially non-specific (10, 11). But in our study, a higher incidence of abdominal pain and hyperamylasemia in the DKA combined with the AP group is observed. We found that patients with combined AP were younger in age, had lower BMI, higher incidence of abdominal pain, and were more likely to be men. From our observations of 25 patients with AP in DKA, the clinical course needs more ICU admission. The mean length of hospital stay was 14 (13.5 and 20) days were longer in the DKA combined with AP groups. No statistical significance in mortality, wherein only one person died which is lower than the previous study (12).

The DAK patients with concurrent AP had higher AG, HCT, RBC, HGB, serum amylase, random glucose, plasma fibrinogen, serum triglyceride, serum cholesterol and HbA1c, lower pH, CO2CP, Ca2+, Na+, and corrected Na+. Some indicators are similar to the previous literature (9). Three risk factors, pH, serum triglyceride, and serum cholesterol, are statistically significant according to the multivariate risk factor analysis. The area under the curves for serum triglyceride, serum cholesterol, and pH were 0.93 (95% CI 0.875–0.985), 0.86 (95% CI 0.768–0.951), and 0.821 (95% CI 0.737–0.905), respectively, for predicting type 2 diabetes DKA patients with AP. The ROC curve analysis was used to identify type 2 diabetic DKA patients with AP with a high negative predictive value based on blood triglycerides of 10.52 mmol/L, serum cholesterol of 9.03 mmol/L, and pH of 7.214.

Detecting AP in DKA patients is often dependent primarily on related symptoms, laboratory results, and radiographic investigations. AP was seen in patients with severe metabolic acidosis characterized by low pH and hyperlipidemia. The triad of hypertriglyceridemia, AP, and DKA has been reported in previous literature (13, 14). Severe hypertriglyceridemia, defined as a serum triglyceride level equal to or more than 1,000 mg/dL, is one of the most prevalent etiologies of acute pancreatitis (AP) (15, 16). In individuals with concomitant diabetic ketoacidosis, it accounts for up to 36.4% of the total (DKA) (9). During DKA episodes, serum triglycerides rise significantly (17). Patients with DKA are in a state of insulin deficiency, which can lead to fat decomposition in adipose tissue during the onset of DKA, and the serum triglyceride is significantly increased. Some studies have proposed the triple sign of DKA, hypertriglyceridemia, and acute pancreatitis, known as the “Enigmatic triangle,” but it is not clear whether DKA is the cause or complication of AP. Insulin deficiency increases lipolysis in adipose tissue, resulting in the release of free fatty acids. Recent studies have suggested that the mechanisms responsible for hypertriglyceridemia-induced AP are related to the accumulation of free fatty acids (FFAs) (18). In this investigation, we discovered that individuals with AP had considerably higher serum triglyceride and cholesterol levels than those without AP. This suggested that hyperlipidemia may be a significant contributor to the pathophysiology of these patients.

Regardless of the combination of DKA and DKA with AP, the severity is assessed by clinical criteria. It is advised to record severity indicators such as APACHE II, SOFA score, and LODS, but there was no difference between the two groups. There had previously been few investigations of DKA worsened by pancreatitis, all of which were described in isolated instances, and even fewer research that used scores to assess illness severity. Only one research found that APACHE II was greater in AP patients with DKA (19).

Emergency physicians face a difficult task in recognizing these concurrent cases and prescribing associated tests to detect them early. By comparing the demographic and clinical characteristics of the two groups on admission, we discovered that more patients in the DKA and AP group were younger in age, and were more men, but they had similar BMI. In comparison to the DKA plus AP group, the PH value is lower, triglyceridemia and cholesterolemia are higher, and the frequently used critical illness score is unable to accurately assess the severity.

We were unable to control all factors in this retrospective analysis, thus certain variables were not compared. Some of the variables that could predict the existence of AP in DKA were not measured using multivariate analysis. The limited sample size constituted a second restriction. In the future, we want to expand our research of DKA with AP by examining a larger sample size prospectively in the hopes of validating our findings and exploring possible risk factors and outcomes.

## Data Availability Statement

The original contributions presented in the study are included in the article/supplementary material, further inquiries can be directed to the corresponding author/s.

## Ethics Statement

The studies involving human participants were reviewed and approved by Beijing Jishuitan Hospital (No. JLKSZD202109-54). The patients/participants provided their written informed consent to participate in this study.

## Author Contributions

LPM and BZ: conception and design. XL and BCC: administrative support. YL and CW: provision of study materials or patients. BZ and CW: collection and assembly of data. LPM, XL, and BCC: data analysis and interpretation. All authors: manuscript writing and final approval of manuscript.

## Conflict of Interest

The authors declare that the research was conducted in the absence of any commercial or financial relationships that could be construed as a potential conflict of interest.

## Publisher's Note

All claims expressed in this article are solely those of the authors and do not necessarily represent those of their affiliated organizations, or those of the publisher, the editors and the reviewers. Any product that may be evaluated in this article, or claim that may be made by its manufacturer, is not guaranteed or endorsed by the publisher.
